# Intra-articular opening wedge osteotomy for varus ankle arthritis with computer-assisted planning and patient-specific surgical guides: a retrospective case series

**DOI:** 10.1186/s12891-022-05437-z

**Published:** 2022-05-21

**Authors:** Xin-long Ma, Jian-xiong Ma, Xing-wen Zhao, Yu-ren Du, Ying Wang, Hao-hao Bai, Bin Lu

**Affiliations:** 1 Tianjin Hospital, Tianjin, 300211 China; 2 Tianjin Key Laboratory of Orthopedic Biomechanics and Medical Engineering, Tianjin Hospital, Tianjin, 300050 China; 3 Graduate School of Tianjin Medical University, Tianjin, 300070 China

**Keywords:** Ankle osteoarthritis, Ankle arthritis, Corrective osteotomy, Preoperative planning, Patient-specific instrument

## Abstract

**Background:**

Computer-assisted preoperative planning, combined with PSI has become an effective technique for treating complex limb deformities. The purpose of this study was to evaluate the efficacy and safety of the novel technique in corrective osteotomy for intra-articular varus ankle deformities associated with osteoarthritis and ankle instability.

**Methods:**

Nineteen patients with intra-articular varus ankle arthritis were reviewed between April 2017 and June 2019, including ten men and nine women with a mean age of 58.3 ± 9.9 years (range, 38 to 76 years). All patients underwent intra-articular opening wedge osteotomy assisted by 3D virtual planning and PSI. Weight-bearing radiographs were used to assess the radiographic results, including TAS angle, TT angle, TMM angle, TC angle, TLS angle, opening-wedge angle, and wedge height. Functional outcomes were assessed by the AOFAS score, VAS score, and ROM of the ankle.

**Results:**

The average follow-up time was 32.2 ± 9.0 months (range, 22 to 47 months). The average union time was 4.4 ± 0.9 months (range, 3.0 to 6.5 months). The TAS angle significantly changed from 84.1 ± 4.6° preoperatively to 87.7 ± 3.1° at the 1-year follow-up and 86.2 ± 2.6° at the latest follow-up. Similarly, the TT angle, TMM angle and TC angle changed significantly at the 1-year follow-up compared with the preoperative assessment and remained stable until the last follow-up. However, the TLS was not corrected significantly. The postoperative obtained opening-wedge angle, and wedge height showed no significant change with preoperative planning. The overall complication rate was 15.8%. The mean VAS score improved from 5.3 ± 0.6 to 2.7 ± 0.7. The mean AOFAS score improved from 56.2 ± 7.6 to 80.6 ± 4.6. However, the ROM showed no significant change.

**Conclusions:**

Accurate correction and satisfactory functional recovery were attained with computer-assisted planning and PSI in the corrective osteotomy of intra-articular varus ankle deformities.

**Supplementary information:**

The online version contains supplementary material available at 10.1186/s12891-022-05437-z.

## Background

Osteoarthritis (OA) is a major global public health problem, with a worldwide prevalence of 23.9% [1], and is characterized by a progressive loss of normal structure and function of the articular cartilage. Compared with knee and hip OA, ankle OA has a lower prevalence, but occurs in patients who are 10 to 15 years younger [2, 3]. Therefore, joint preservation instead of total ankle replacement or ankle arthrodesis is very important to prolong ankle function, especially for young and active patients.

Supramalleolar osteotomy (SMO) is a well-established technique to realign the mechanical axis of the limb and redistribute the loads on the ankle joint, thereby improving the biomechanics of the joint and preventing further degeneration [4–9]. Nevertheless, in some cases, the apex of deformity, i.e. the center of rotation and angulation (CORA) may be located within the joint, and translation of the distal fragment occurs because the correction is not performed at the CORA using traditional SMO [2]. In addition, persistence of the medial intra-articular defect may result in recurrent varus deformities after extra-articular osteotomy [10, 11]. Thereby, Becker et al.[12] described an intra-articular opening medial tibial wedge osteotomy, known as plafond-plasty, which avoids creating a secondary translational deformity due to the osteotomy level and hinge is intentionally made at the CORA. Although several studies have reported satisfactory results of this procedure [10–14], there are still some challenges in actual operation. First, conventional X-ray radiographs cannot provide 3-dimensional (3D) quantification of multiplanar complex deformities. In addition, precise reproduction of the surgical plan remains challenging with freehand operation.

Recently, the combination of computer-assisted planning and patient-specific instruments (PSIs) has been reported as a mature technology to achieve accurate correction of complex limb deformities [15–20]. We further extended this technique in the correction of ankle OA deformities, and the purpose of this study was to verify the reliability and accuracy of PSI in corrective osteotomy for intra-articular varus OA with medial distal tibial platform erosion.

## Methods

### Ethical considerations

Ethical approval for the retrospective study was obtained from the local ethics committee of Tianjin hospital. Informed consent was obtained from all enrolled patients.

### Patient selection

Patient inclusion criteria were as follows: (1) symptomatic varus ankle OA undergoing at least 1-year of conservative treatment; (2) ankle OA associated with medial distal tibial platform erosion; (3) normal lateral tibial surface. The exclusion criteria were: (1) rigid deformity; (2) infective arthritis or rheumatoid arthritis; (3) patients with neuropathic arthropathy or rheumatoid arthritis. In total, a consecutive series of 19 patients (19 ankles, 9 left and 10 right) who underwent PSI assisted ankle plafond-plasty associated with varus ankle OA were enrolled between April 2017 and June 2019. There were 10 men and 9 women enrolled, with a mean age of 58.3 ± 9.9 years (range, 38 to 76 years), and a mean body mass index (BMI) of 25.9 ± 2.4 kg/m^2^ (range, 20.3 to 29.8 kg/m^2^). Three cases (15.8%) were bilateral ankle osteoarthritis, but only unilateral correction was performed this time. Ten ankles (52.6%) were Takakura stage II, 7 ankles (36.8%) were Takakura stage IIIa, and 2 ankles (10.5%) were Takakura stage IIIb. A summary of the demographics is presented in Table 1.

**Table 1:** Demographic characteristics, surgical procedures and adverse events

Case/Gender/Side	Age (yrs)	BMI (kg/m^2^)	Takakura stage	Planned correction (°)		F/U (mo)	Time to Union (mo)	Additional surgical procedures	Adverse events
				**TAS**	**TLS**				
1/M/R	55	27.3	IIIa	87	74	46	4	Osteophyte debridement	-
2/M/R	65	29.4	IIIa	86	73	47	4	Osteophyte debridement, lateral ligament reconstruction, deltoid ligament release	-
3/F/L	46	26.9	IIIa	84	84	44	5	Osteophyte debridement, lateral ligament reconstruction	Hinge fracture
4/ F/R	47	22.4	II	85	80	43	5.5	Osteophyte debridement, lateral ligament reconstruction	Hinge fracture
5/ F/R	66	27.1	II	89	77	40	4	Osteophyte debridement	-
6/M/L	64	24.2	IIIb	90	80	40	4	Osteophyte debridement, , deltoid ligament release	-
7/M/L	38	26.5	IIIa	85	80	37	6.5	Osteophyte debridement	Hinge fracture
8/F/L	54	24.8	IIIa	85	80	33	3.5	Osteophyte debridement	-
9/M/R	67	20.3	II	83	78	33	4	Osteophyte debridement, posterior tibial tendon transferring, Achilles tendon lengthening	-
10/F/L	62	22.9	IIIb	88	82	32	6	Osteophyte debridement, lateral ligament reconstruction	-
11/F/L	54	23.4	IIIa	84	80	29	4	Osteophyte debridement, deltoid ligament release	-
12/M/R	76	27.7	IIIa	88	80	25	3.5	Osteophyte debridement	-
13/M/R	67	25.5	II	88	78	22	3	Osteophyte debridement, lateral ligament reconstruction	-
14/M/L	49	29.8	II	90	80	23	4	Osteophyte debridement, posterior tibial tendon transferring	-
15/F/L	67	26.1	II	90	79	22	4	Osteophyte debridement, lateral ligament reconstruction	-
16/M/R	48	25.8	II	88	81	23	4	Osteophyte debridement, deltoid ligament release	-
17/F/L	67	27.3	II	86	80	22	5	Osteophyte debridement, lateral ligament reconstruction	-
18/F/R	63	26.7	II	87	82	26	4	Osteophyte debridement, lateral ligament reconstruction	-
19/M/R	52	27.8	II	88	78	24	5	Osteophyte debridement, lateral ligament reconstruction	-
Mean (SD)	58.3 (9.9)	25.9 (2.4)	-	86.9 (2.2)	79.3 (2.6)	32.2 (9.0)	4.4 (0.9)	-	-

### Preoperative planning

Before preoperative planning, a physical examination was performed to assess the lower limb alignment, heel varus or valgus, range of motion (ROM) and stability of the ankle joint. Weight-bearing anteroposterior and lateral radiographs were taken to evaluate the overall mechanical axis, and to design a preliminary correction plan, according to the method described by Paley [21].

**Fig. 1:**
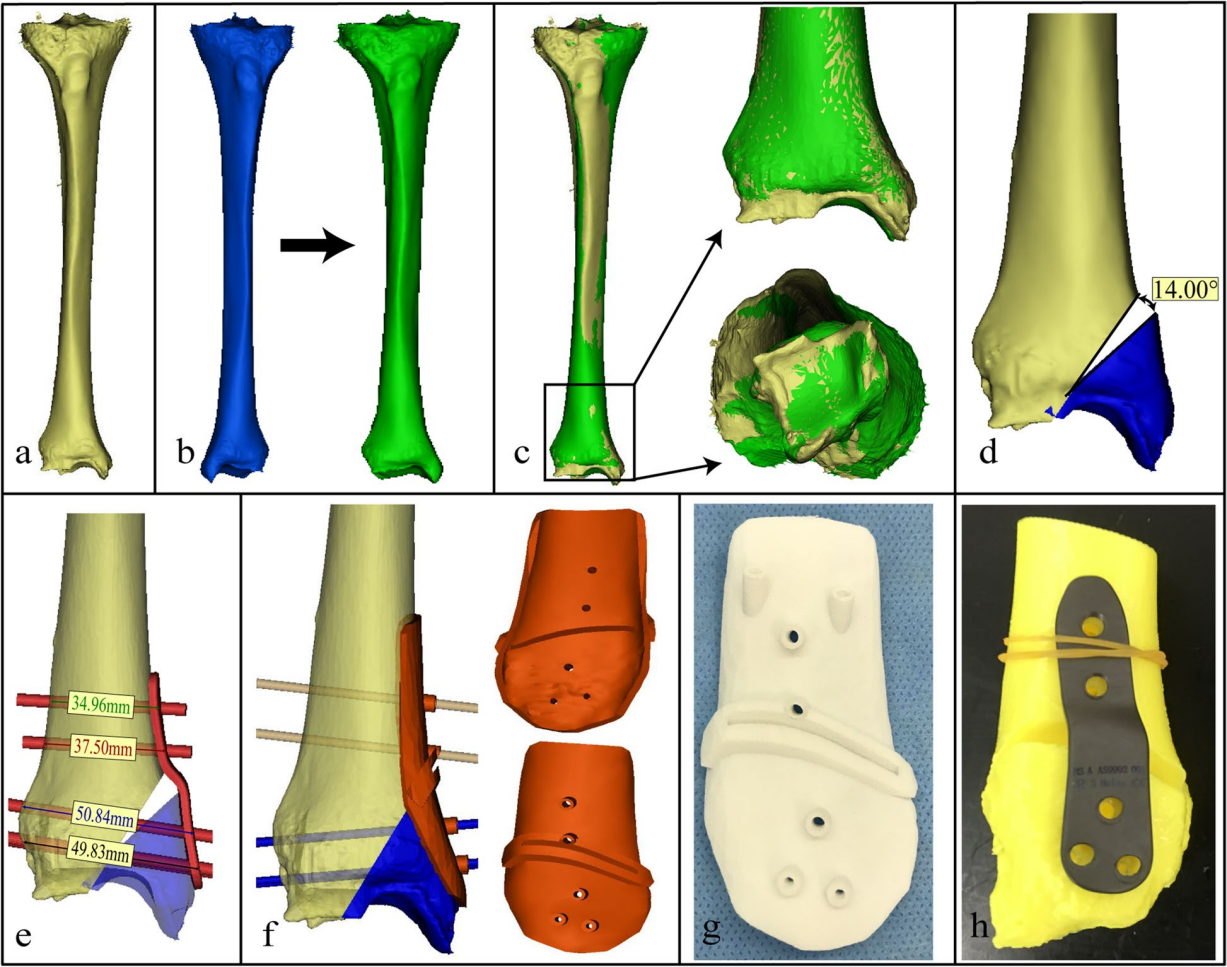
Workflow of computer-assisted planning and patient-specific surgical guide design (right ankle case 2). **a** The 3D model of affected bone (right, yellow). **b** The 3D model of unaffected bone (left, blue) and the mirror model of the left normal bone (green). **c** The affected bone was superimposed over the mirror model of the left normal bone. **d** An opening wedge osteotomy was simulated. **e** A patient-matched plate was designed with the lengths and trajectories of every screw marked. **f** The final designed patient-specific guide that fits the surface of the tibia. **g**-**h** The physical model of the guide, the patient-matched plate and tibia

Subsequently, all patients underwent a CT scan (GE LightSpeed CT scanner, Wisconsin, USA) of the deformed and contralateral healthy ankle joints containing the entire tibia, fibula, and foot with a low-dose setting (scan pitch, 1.375:1; tube current, 90 mA; tube voltage, 40 kV). Then the CT data were exported into Mimics 22.0 (Materialise MV, Belgium) in DICOM format, and 3D models of both lower limbs were constructed. In this step, if the contralateral tibia had normal morphology and alignment, it would be mirrored as a reference bone and the proximal nondeformed region of the affected tibia model was superimposed with the mirrored reference bone (Fig. 1a-c). Thus, the amount of 3D deformity of the distal part of the tibia and tibial plafond was determined, as well as the correction angle. Next, an osteotomy plane, perpendicular to the frontal plane, was created on the CORA. Then, the osteotomy was performed, and the fragment was virtually reduced to the correction position by aligning it to the resembling part of the reference bone after the osteotomy plane, hinge position, and sawing direction were identified by the surgeon (Fig. 1d). When both sides were pathologics, osteotomy and reduction were planned according to the reference values reported in the literature (TAS was set as 90°; TLS was set as 80°) [21]. Next, a patient-matched plate, which was designed to fit the bone surface after the corrective osteotomy, was aligned to the planned position with every trajectory of the screw marked by cylinders and the lengths of screws and depth of the cutting were measured to serve as a reference for actual surgery (Fig. 1e). Then the fragment, together with the marked cylinders and the osteotomy plane, was restored to its original deformed position. In addition, three or more additional cylinders were introduced parallel to the tibial plafond within the subchondral bone just under the articular cartilage [13], and a surgical guide was designed according to the individual anatomical surface of the affected bone with a cut slot and several drill cylinders were added. Finally, the surgical guide was shaped to fit the bone surface with osteotomy slots to direct the chisels or saws, and predrilled holes for fixing the guide on the bone as well as matching the plate’s screw positions during surgery. In particular, the distal holes were used to guide the insertion of Kirschner wires to prevent penetration of the saw blade into the joint, and to act as a hinge during correction (Fig. 1f).

Afterwards, the physical models of bone, and guide were manufactured by nylon with 3D printing technology (SHILUOKE MEDICAL, China) to preoperatively simulate the osteotomy, reduction, and other specific operative steps. Patient-matched plates were made in TI6AL4V alloy (WASTOM medical, China). Then sterilization was performed using the conventional steam pressure method (Fig. 1g-h).

### Surgical procedures

**Fig. 2:**
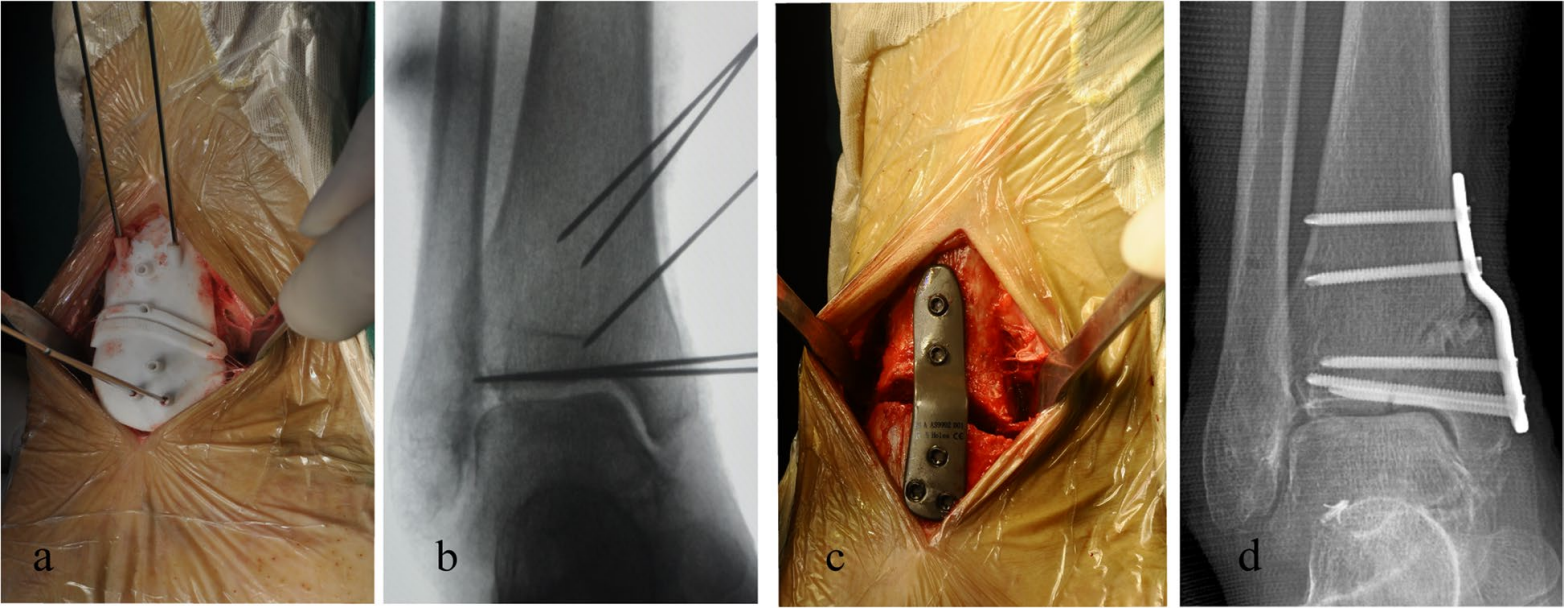
Intraoperative application of patient-specific surgical guides and patient-matched plates (right ankle case 2). **a**-**b** The guide was intraoperatively placed onto the planned surgical position and checked by fluoroscopy, and osteotomy was completed with a bone saw through the slot on the guide. **c**-**d** Correction and fixation were completed with the patient-matched plate by inserting locking screws into the predrilled holes

All surgical procedures were completed under general anaesthesia by a single experienced surgeon. The osteotomy approach was performed through a 5-cm medial longitudinal incision. After the osteotomy targeting position was adequately exposed as a preoperative simulation, the PSI was placed on the tibial surface, and fixed with Kirschner wires through the sleeves in the PSI. Then, a fluoroscopy check was performed to identify the position of the guide as well as the osteotomy plane (Fig. 2a-b). Afterwards, the intra-articular osteotomy was performed carefully with an oscillating saw under the guidance of the cutting slot. The sawing depth was monitored by the length scale of the saw blade with referring to the previously measured results in the preoperative virtual simulation plan. The guide was subsequently removed, but the Kirschner wires remained on the bone, especially the distal three Kirschner wires, which were inserted within the subchondral bone just under the articular cartilage at the apex of the plafond. An osteotome was then inserted gradually to complete the osteotomy, and the cortical gap was carefully and slowly opened by serial insertion of another osteotome until the Kirschner wires matched the screw holes in the plate, and the distracted wedge was maintained by a lamina spreader. Finally, correction and fixation were automatically achieved by placing the patient-matched plate and inserting screws into the predrilled holes. Then the plate position, and ankle alignment and position in the mortise were checked with fluoroscopy (Fig. 2c-d).

After adequate fixation of the distal tibia, the autologous cortical iliac bone or allograft was packed into the osteotomy gap. In addition, for the cases that presented with contraction of the medial structure or Achilles tendon, a deltoid ligament release and Achilles tendon lengthening were performed. If there was incompetence of the peroneus brevis tendon, a tendon transferring procedure was performed. Finally, an osteophyte debridement procedure was performed in the patients with an osteophyte > 2 mm on the talar neck and anterior fibula. However, it was retained on the tibial side in order to prevent destabilization of the ankle. All additional procedures are summarized in Table 1.

### Postoperative management and follow-up

The ankle joint was fixed in neutral posture by a short leg cast immediately after surgery, then, a newly shaped cast or a removable boot was applied for 6 to 8 weeks when wound healing was appropriate. Continuous range of motion exercises without weight-bearing were performed for the first 3–6 weeks after surgery, followed by touchdown weight-bearing. Full weight-bearing without a plaster cast was recommended when adequate radiographic bony union was achieved. Patients were regularly viewed at 1 month, 2 months, 3 months, 6 months, and 1 year, and then annually.

### Radiographic assessment

**Fig. 3:**
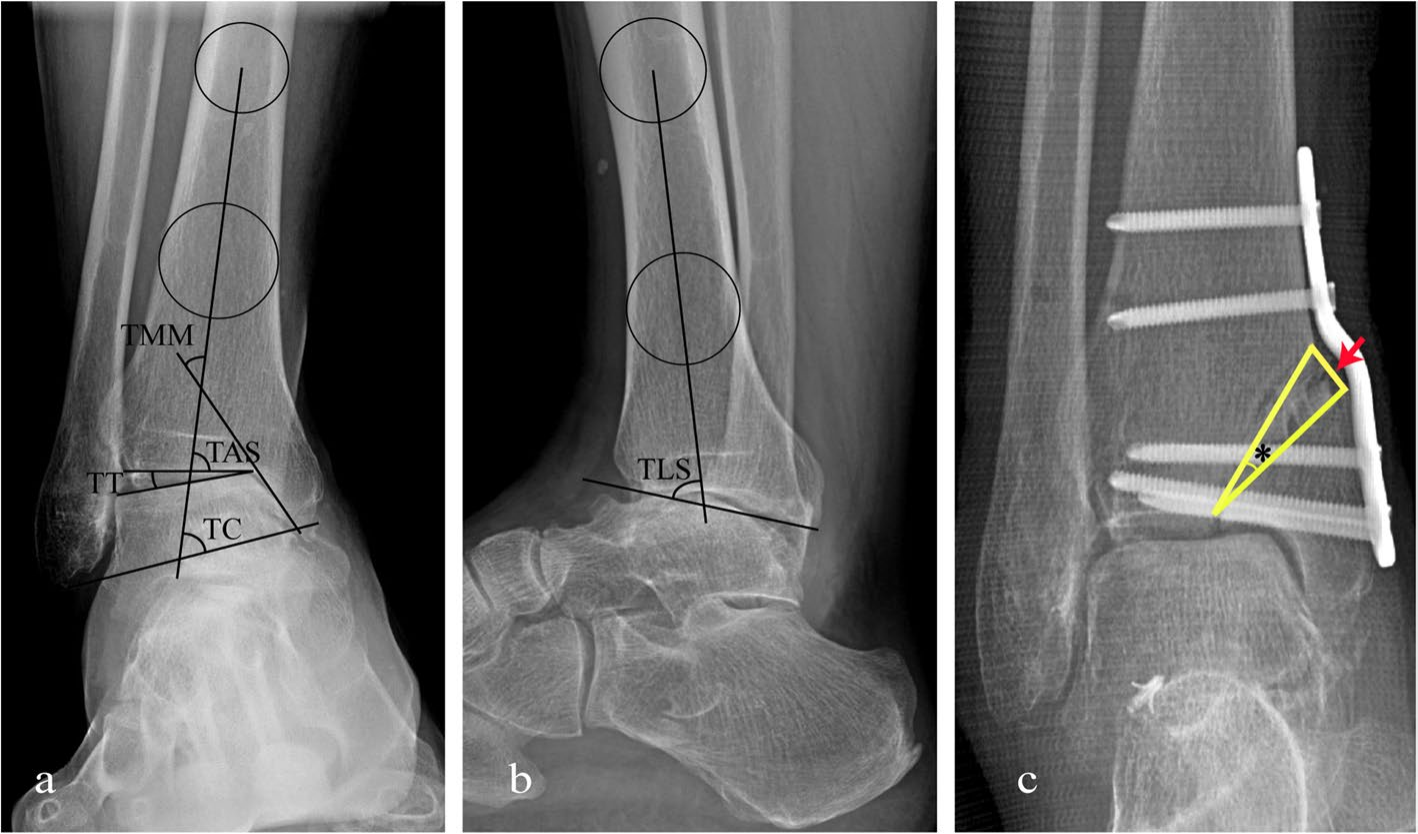
Schematic measurements for radiographic parameters in anteroposterior and lateral weight bearing X-rays of the ankle joint. **a** TAS angle was defined as the angle between the axis of the tibia and the distal articular surface of the tibia; TT angle was defined as the angle between the distal articular surface of the tibia and the upper surface of the talus; TMM angle was defined as the angle between the axis of the tibia and the articular surface of medial malleolus; TC angle was defined as the angle between the axis of the tibia and a line drawn from the apex of medial malleolus to the apex of lateral malleolus. **b** TLS angle was defined as the angle between the axis of the tibia and the surface of the distal tibia in the lateral view. **c** The opening-wedge angle was defined as the angle between the upper and lower osteotomy margins after distraction (asterisk), and the wedge height was defined as the distance between the upper and lower osteotomy margins on the medial tibial cortex (red arrow)

Preoperatively and at every follow-up visit, all radiographic parameters were measured on weight-bearing anteroposterior and lateral ankle radiographs, including the tibial anterior surface angle (TAS), the talar tilt angle (TT), the tibial medial malleolus angle (TMM), the talocrural angle (TC), and the tibial lateral surface angle (TLS) (Fig. 3a-b). Additionally, the preoperative and postoperative opening-wedge angle and wedge height were measured on weight-bearing anteroposterior radiographs to assess the accuracy of correction (Fig. 3c). Osteoarthritis grading was graded according to the Takakura classification [22]. Osteotomy gap union was defined as satisfying clinical criteria (no pain, no warmth, improvement in swelling, and stability to stress) and radiographic criteria (visible trabecular bridging across the osteotomy site and no lucency around the hardware) [11].

### Clinical assessment

A physical examination and standardized questionnaire were used preoperatively and during postoperative follow-ups to assess the clinical outcomes. The ankle range of motion (ROM) was measured with a manual goniometer applied along the lateral border of the leg and foot. Pain of the ankle during walking was evaluated with the visual analogue scale (VAS) ranging from 0 (no pain) to 10 points (maximal pain). The American Orthopaedic Foot and Ankle Society (AOFAS) hindfoot score was recorded to assess the preoperative and postoperative functional changes. Adverse events were also documented, including hinge fractures, nerve injury, infection, skin necrosis, delayed union or nonunion, and malunion.

### Statistical analysis

The mean and standard deviation of all data were calculated and analysed using SPSS Statistics version 22.0 (IBM, New York, USA). Preoperative, 1 year follow-up postoperative, and the most recent follow-up radiographic results were compared. All radiographic parameters were measured by two independent orthopaedic surgeons, who were not involved in the treatment. All 19 cases were re-evaluated by the same observer 2 months after the initial measurement. The intraclass correlation coefficients (ICCs) were used to assess the interobserver and intraobserver reliability (Table S1), and the mean value (with the exclusion of the maximum and the minimum) was recorded. Preoperative and the most recent follow-up clinical results were compared. The paired Student *t* test was used to compare the preoperative and follow-up investigations if the data were confirmed to be normally distributed using the Kolmogorov–Smirnov test. Otherwise the nonparametric Wilcoxon signed-rank test was performed. A *P* value < 0.05 was considered to be statistically significant.

## Results

### Demographics

All patients were successfully stabilized with an individualized plate. Other associated procedures included osteophyte debridement in 19 ankles, lateral ligament reconstruction in 9 ankles (47.4%), Achilles tendon lengthening in case 9 (5.3%), and posterior tibial tendon transfer in case 9 and case 14 (10.5%). Among the 9 cases with lateral ligament reconstruction, cases 2 and 3 were repaired with a peroneus brevis tendon by a modified Chrisman Snook procedure, and the others were reinforced by imbrication with the inferior extensor retinaculum using suture anchors. There was no loss of follow-up. The mean follow-up duration was 32.2 ± 9.0 months (range, 22 to 47 months). The mean union time was 4.4 ± 0.9 months (range, 3.0 to 6.5 months) (Table 1).

### Radiological outcomes

The mean TAS significantly changed from 84.1 ± 4.6° (range, 70.7°—91.2°) preoperatively to 87.7 ± 3.1° (range, 81.7°—92.7°) at the 1-year follow-up (*P* < 0.001), and to 86.2 ± 2.6° (range, 81.2°—90.4°) at the latest follow-up (*P* = 0.045). The mean TT significantly changed from 15.5 ± 6.4° (range, 4.6°—29.3°) preoperatively to 5.9 ± 4.3° (range, 0°—16.1°) at the 1-year follow-up (*P* < 0.001), and to 5.0 ± 2.9° (range, 1.1°—9.7°) at the latest follow-up (*P* < 0.001). Similarly, the mean TMM and TC changed significantly at the 1-year follow-up compared with the preoperative assessment and remained stable until the last follow-up (Table 2). However, the TLS was not corrected significantly (*P* > 0.05). In terms of the accuracy of correction, the opening-wedge angle and wedge height showed no significant change when compared preoperatively and postoperatively (*P* > 0.05).

**Table 2:** The comparison of preoperative and postoperative function outcomes and limb alignment

**Parameter**	**Numerical Values**^a^	***P* value**^b^
**TAS (°)**		
Preoperative	84.1 ± 4.6 (70.7—91.2)	
1 year post-op	87.7 ± 3.1 (81.7—92.7)	0.001
Last F/U	86.2 ± 2.6 (81.2—90.4)	0.045
**TT (°)**		
Preoperative	15.5 ± 6.4 (4.6—29.3)	
1 year post-op	5.9 ± 4.3 (0—16.1)	< 0.001
Last F/U	5.0 ± 2.9 (1.1—9.7)	< 0.001
**TMM (°)**		
Preoperative	33.8 ± 6.8 (23.1—47.9)	
1 year post-op	28.9 ± 6.3 (17.0—40.2)	0.015
Last F/U	28.8 ± 5.8 (19.7—37.9)	0.002
**TC (°)**		
Preoperative	72.9 ± 4.3 (65.3—81.0)	
1 year post-op	79.3 ± 5.5 (71.8—90.1)	< 0.001
Last F/U	78.9 ± 4.5 (71.1—88.7)	< 0.001
**TLS (°)**		
Preoperative	79.5 ± 7.3 (65.6—94.29)	
1 year post-op	80.2 ± 5.5 (71.2—90.6)	0.544
Last F/U	79.8 ± 4.2 (73.3—86.8)	0.839
**Opening-wedge angle (°)**		
Planned	16.1 ± 5.7 (7.5—29.3)	
Obtained post-op	15.7 ± 5.3 (7.9—29.1)	0.904^c^
**Wedge height (mm)**		
Planned	8.0 ± 3.2 (3.9—17.3)	
Obtained post-op	8.1 ± 3.3 (4.0—17.6)	0.165^c^
**ROM (°)**		
Preoperative	32.9 ± 4.2 (25.0—40.0)	
Last F/U	34.5 ± 4.4 (30.0—40.0)	0.069^c^
**VAS at gait**		
Preoperative	5.3 ± 0.6 (4.0—6.0)	
Last F/U	2.7 ± 0.7 (2.0—4.0)	< 0.001^c^
**AOFAS scores**		
Preoperative	56.2 ± 7.6 (45.0—74.0)	
Last F/U	80.6 ± 4.6 (73.0—89.0)	< 0.001

^a^The values are given as the mean ± SD (range)

^b^The *P* values shown are for the comparisons between the preoperative and follow-up investigations

^c^Wilcoxon signed-rank test

### Clinical and functional outcomes

The mean ROM of the ankle improved from 32.9 ± 4.2° (range, 25.0°—40.0°) preoperatively to 34.5 ± 4.4° (range, 30.0°—40.0°) postoperatively, but this finding was not statistically significant (*P* = 0.069). The mean VAS score significantly improved from 5.3 ± 0.6 (range, 4.0—6.0) preoperatively to 2.7 ± 0.7 (range, 2.0—4.0) at the latest follow-up (*P* < 0.001). Similarly, the AOFAS score that increased significantly from 56.2 ± 7.6 (range, 45.0—74.0) to 80.6 ± 4.6 (range, 73.0—89.0) at the latest follow-up (*P* < 0.001). The clinical outcomes are summarized in Table 2.

**Fig. 4:**
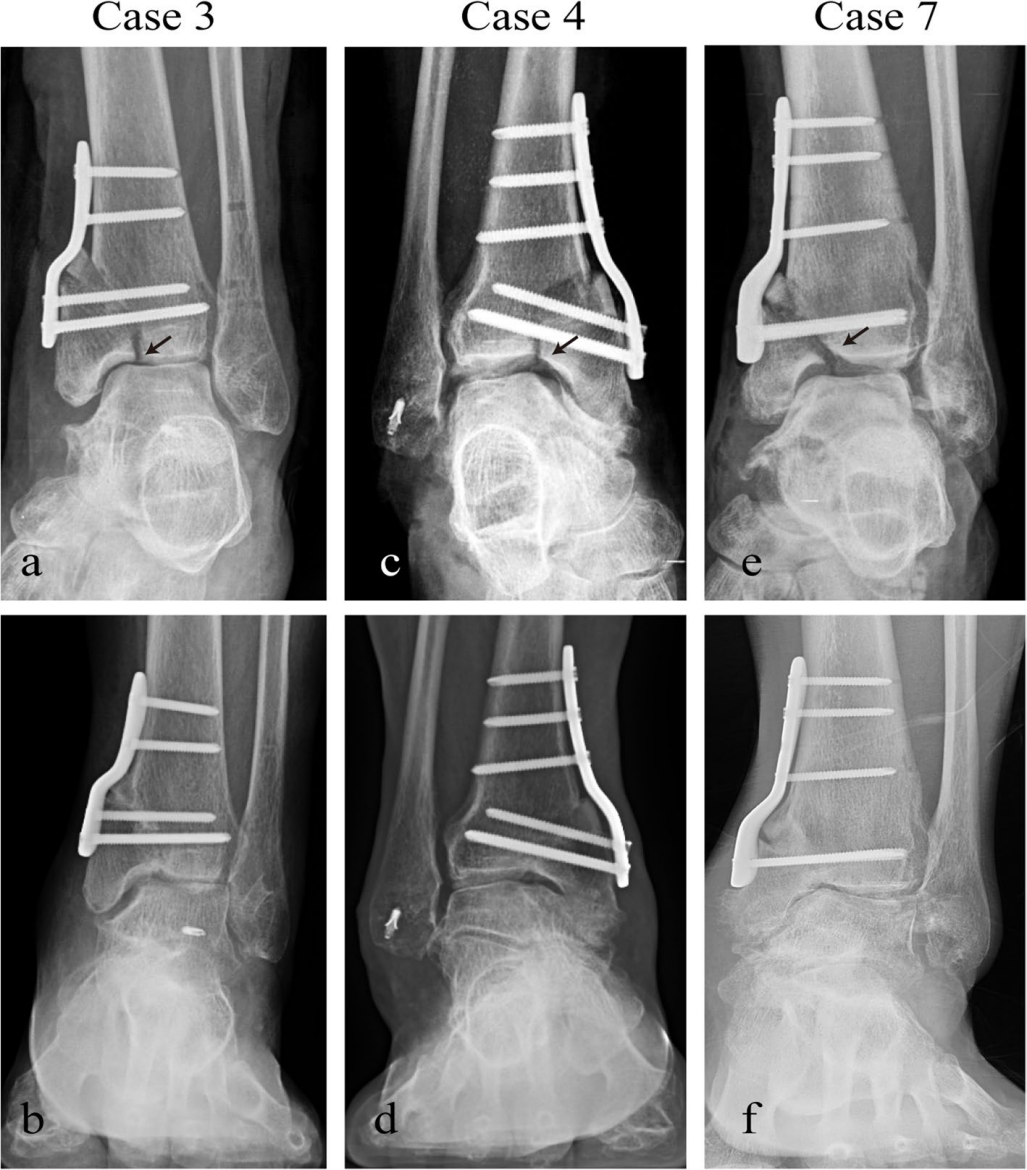
Three cases with intraoperative hinge fractures are indicated by the arrows. Callus formation was observed at 5 months **a**-**b** 5.5 months. **c**-**d** and 6.5 months **e**–**f** after surgery

Except for three intraoperative hinge fractures (15.8%), there was no wound infection, skin necrosis, nonunion, nerve injury, or other complications observed. However, no obvious displacement occurred with nonweight bearing treatment for 12 weeks after surgery, and fracture union was obtained within a mean of 5.7 months after surgery (range, 5 – 6.5 months) without additional osteosynthesis surgery (Fig. 4). The hardware of case 1 and case 2 was removed at 18 months and 15 months after the initial surgery, respectively, because of personal reasons. No patients to date have required conversion to ankle arthrodesis or ankle arthroplasty due to progression of ankle arthritis or worsening symptoms (Table 1).

## Discussion

The most important finding of this study is that performing plafond-plasty with 3D virtual planning and PSI is a reliable and accurate procedure.

Plafond-plasty is an intra-articular opening medial tibial wedge osteotomy, that is designed to correct varus ankle deformities with medial distal tibial platform erosion. Mann et al. [13] reported that varus ankle tilt significantly improved by 8°, and the AOFAS score significantly improved by 32 points in 19 ankles with this technique. In this study, they confirmed that the overall outcome of intra-articular plafond-plasty is very good in terms of pain relief, correction of any existing mechanical malalignment, and slowing progression of arthritic changes in the ankle joint [13]. Xu et al. [10] subsequently reported that plafond-plasty combined with lateral ligament reconstruction is an effective method to treat stage IIIB ankle deformities. Pain relief, and functional improvement with this procedure may be explained by the improvement in ankle gait biomechanics after the realignment of the joint axis. In addition, the improvement of ankle stability, and the redistribution of forces on the ankle joint after osteotomy are beneficial for restoring congruency of the ankle joint, and repairing articular defects [23]. However, the obvious shortcomings of these studies are that the preoperative diagnosis and planning relied on plain radiographs and the surgeon’s experience. First, the X-ray radiographs could not provide the 3D quantification of multiplanar complex deformities. The anatomical axis of the tibia and angles of the ankle, which were drawn by freehand, may be influenced by the inhomogeneous magnification rate of the radiographs and the posture and position of the limb during fluoroscopy. Moreover, only target values of TAS and TLS could be designed in the preoperative plan. In addition, accurate replication of this plan at the time of surgery is difficult, and heavily depends on the surgical skills and experience of the surgeons. In particular, in reference to our own experience, accurately inserting distal parallel Kirschner wires into the impacted portion of the tibial plafond is high technically demanding in actual operations but necessary, since it keeps the distal subchondral bone bridge intact and ensures the exact position of the correction hinge.

To date, studies on the application of computer-assisted 3D virtual planning and PSI in treating complex deformities of the foot and ankle have been investigated less, however, the results are promising [15–17, 20]. Weigelt et al. [15] reported that postoperative CT scans showed accurate reduction with a residual translational and rotational error of < 3 mm and < 6° in 4 cases who underwent computer-assisted corrective osteotomy of ankle deformities. A recent study compared the results of this technique with those of conventional methods in ankle surgery [20]. In this study, 11 ankles who underwent PSI assisted SMO showed a significantly shorter mean operating time and postoperative hospital stay, a decreased number of fluoroscopy examinations than conventional surgery, and better clinical and radiographic results. The authors pointed out that with the help of computer-assisted surgery planning, we can not only thoroughly understand the complex deformities of the ankle, but also make precise diagnoses and even design personalized treatment plans for individuals. In addition, the preoperative plan is reproduced by the application of patient-specific surgical guides during actual surgery [15, 20].

In the present study, we introduced the application of computer-assisted planning and PSI in corrective osteotomy of intra-articular varus ankle deformities. As a result, all cases obtained satisfactory improvement of VAS, and AOFAS with no serious complications occurred at a mean follow-up period of 32.5 months. In addition, the weight bearing radiographic parameters, including TAS, TT, TMM, and TC also significantly improved when compared with the preoperative values. These results were in accordance with previous studies, and confirmed that meticulous preoperative planning of deformity correction and limb realignment is essential to help redistribute joint forces within the ankle and to prevent the development of arthrosis or halting its progression [2, 3, 13, 20]. In addition, postoperative radiographic results showed accurate osteotomy and reduction with an error of opening-wedge angle and wedge height < 0.9° and < 0.3 mm, respectively, when compared with the values of preoperative planning. According to the feedback of the surgeons, the procedures of the insertion of distal subchondral bone Kirschner wires, osteotomy, and reduction were simplified with the use of PSI. Furthermore, our technique was different from the others in that we took the implant into account during design procedures, therefore, the plate could act as a reduction guide, and the placement of the implant and the insertion of locking screws could be simultaneously guided by the PSI with no need for an additional reduction guide in the operation. In addition to the benefits mentioned, we printed real-scale models for preoperative simulation and intraoperative reference, which provided both tactile and visual feedback [16, 24]. This technique also enhanced the surgeon’s understanding of the deformity and subsequent surgery, and provided practice opportunities for the residents and training fellows [16]. Finally, it enabled us to preoperatively verify the position of the implants, and design the shape of the bone graft to increase the accuracy of certain procedures.

In our study, no intraoperative tendon or neurovascular structure injuries were recorded. This may be related to the application of PSI, which accurately guided the osteotomy orientation, and cutting depth during surgery. However, 3 cases of hinge fractures were observed. All of them obtained bone union within 5.7 months and achieved satisfactory results at the final follow-up. Although it was not mentioned in previous reports, we believed it to be a common but minor complication in intra-articular osteotomy because a similar fracture pattern was also found in the cases in the current literature [10, 11]. In our experience, the main cause may be related to the surgical skills. If the bone graft was inserted prior to plate fixation, the intact subchondral bone hinge may be disrupted after the bone stock was pushed into the defect, especially when operating with violence or when the shape of the graft was not well shaped. Therefore, fixation before bone grafting, or an individualized bone graft may be essential to prevent such adverse events. Additionally, common postoperative complications, such as wound healing problems, and malunion or nonunion at the osteotomy site were not observed in our study. Wound healing was obtained within 2 weeks, and osteotomy union was observed in all patients within 4.4 months after the surgery without loss of correction or implant failure in the present study. This may be related to the application of a patient-matched plate, which was compatible with the irregular surface of bones, and minimized incision tension, as well as skin irritation. In addition, the superior mechanical properties of patient-matched plates could reduce the risk of plate fracture, screw loosening, and bone resorption [17].

In accordance with previous studies, we found that bony correction alone is not enough to correct varus ankle deformities and maintain the stability of the ankle associated with asymmetric osteoarthritis [13]. Therefore, 11 of 19 cases in our study underwent additional soft tissue reconstruction procedures, and acquired good clinical results. In addition, plafond-plasty only improved the alignment in the coronal plane, and did not help with correction in the sagittal plane, which can be indicated by the unchanged TLS angle in our study. Moreover, CT-reconstructed 3D models showed that the erosion of the tibial plafond is dome shaped and that the intra-articular deformity is multiplanar. Therefore, the indications for intra-articular osteotomy correction still need to be further defined. Perhapse additional fibular osteotomy, calcaneal osteotomy, and even an extra-articular osteotomy are necessary to achieve 2-plane correction, and restore tibiotalar joint congruency [2, 3, 11].

Numerous limitations should be noted in the present study. First, a cohort with 19 ankles was relatively small and there was no control group, therefore it was unclear whether the new technique is superior to other procedures such as manual osteotomy or computer-navigated osteotomy. In addition, the mean duration of follow-up was limited to 32.2 months, which was relatively short. A larger sample and longer follow-up prospective clinical studies are needed in the future to verify the advantages and disadvantages of this technique. Second, extra radiation exposure was increased because the preoperative CT scans of the bilateral limbs were necessary to perform the 3D planning and PSI design. However, the exposure level of intraoperative fluoroscopy is approximately 20–200 mSv per minute [25], but only 0.07 mSv for the CT scanning of ankle [26], and it would be decline even further by reducing the volume areas [27]. Furthermore, the decrease in intraoperative use of the C-arm with the application of virtual simulation and PSI was confirmed [20, 28], as well as the simplification of surgical procedures and reducing in surgical time [15, 16, 28], therefore, we believe that the benefits outweigh the injuries. Another limitation was the time and economic cost associated with 3D design and printing. It would take approximately 4 to 7 days from obtaining the necessary image studies to the printing of models, and the average expense of PSI is approximately 600 dollars, however, it may gradually decrease with the rapid development of 3D technology, and the improvement in health care policies, and an increasing number of professionals are engaged in this work.

## Conclusions

In conclusion, accurate correction and satisfactory functional recovery were attained with computer-assisted planning and PSI in the corrective osteotomy of intra-articular varus ankle deformities.

## Supplementary Information

**Additional file 1:** **Table S1** The statistics for inter-observer reliability and intra-observer reproducibility

## Data Availability

The datasets used and/or analysed during the current study available from the corresponding author on reasonable request.

## Abbreviations

OA: Osteoarthritis
SMO: Supramalleolar osteotomy
CORA: The center of rotation and angulation
3D: Three-dimension
PSI: Patient-specific instruments
VAS: Visual analogue scale
AOFAS: The American Orthopaedic Foot and Ankle Society Ankle-Hindfoot scores
BMI: Body mass index
TAS: The tibial anterior surface angle
TT: The talar tilt angle
TMM: The tibial medial malleolus angle
TC: The talocrural angle
TLS: The tibial lateral surface angle
ICC: Intraclass correlation coefficients
SD: Standard deviation
F/U: Follow-up

## References

[1] Paterson KL, Gates L (2019). Clinical Assessment and Management of Foot and Ankle Osteoarthritis: A Review of Current Evidence and Focus on Pharmacological Treatment. Drugs Aging.

[2] Knupp M (2017). The Use of Osteotomies in the Treatment of Asymmetric Ankle Joint Arthritis. Foot Ankle Int.

[3] Hintermann B, Knupp M, Barg A (2016). Supramalleolar Osteotomies for the Treatment of Ankle Arthritis. J Am Acad Orthop Surg.

[4] Krähenbühl N, Akkaya M, Deforth M, Zwicky L, Barg A, Hintermann B (2019). Extraarticular Supramalleolar Osteotomy in Asymmetric Varus Ankle Osteoarthritis. Foot Ankle Int.

[5] Zhao H, Wen X, Zhang Y, Liang J, Liu P, Li Y, Lu J, Liang X (2019). Supramalleolar osteotomy with medial distraction arthroplasty for ankle osteoarthritis with talar tilt. J Orthop Surg Res.

[6] Krähenbühl N, Zwicky L, Bolliger L, Schädelin S, Hintermann B, Knupp M (2017). Mid- to Long-term Results of Supramalleolar Osteotomy. Foot Ankle Int.

[7] Jung H, Lee D, Lee S, Eom J (2017). Second-Look Arthroscopic Evaluation and Clinical Outcome After Supramalleolar Osteotomy for Medial Compartment Ankle Osteoarthritis. Foot Ankle Int.

[8] Ahn T, Yi Y, Cho J, Lee W (2015). A Cohort Study of Patients Undergoing Distal Tibial Osteotomy without Fibular Osteotomy for Medial Ankle Arthritis with Mortise Widening. J Bone Joint Surg Am.

[9] Nüesch C, Huber C, Paul J, Henninger HB, Pagenstert G, Valderrabano V, Barg A (2015). Mid- to Long-term Clinical Outcome and Gait Biomechanics After Realignment Surgery in Asymmetric Ankle Osteoarthritis. Foot Ankle Int.

[10] Xu Y, Li X, Guo C, Xu X (2021). Intra-articular opening osteotomy combined with lateral ligament reconstruction for varus ankle arthritis. J Orthop Surg Res.

[11] Hintermann B, Ruiz R, Barg A (2017). Novel Double Osteotomy Technique of Distal Tibia for Correction of Asymmetric Varus Osteoarthritic Ankle. Foot Ankle Int.

[12] Becker AS, Myerson MS (2009). The Indications and Technique of Supramalleolar Osteotomy. Foot Ankle Clin.

[13] Mann HA, Filippi J, Myerson MS (2012). Intra-Articular Opening Medial Tibial Wedge Osteotomy (Plafond-Plasty) for the Treatment of Intra-Articular Varus Ankle Arthritis and Instability. Foot Ankle Int.

[14] Myerson MS, Zide JR (2013). Management of Varus Ankle Osteoarthritis with Joint-Preserving Osteotomy. Foot Ankle Clin.

[15] Weigelt L, Fürnstahl P, Hirsiger S, Vlachopoulos L, Espinosa N, Wirth SH (2017). Three-Dimensional Correction of Complex Ankle Deformities With Computer-Assisted Planning and Patient-Specific Surgical Guides. J Foot Ankle Surg.

[16] Fillat-Gomà F, Marcano-Fernández FA, Coderch-Navarro S, Martínez-Carreres L, Berenguer A (2021). 3D printing innovation: New insights into upper extremity surgery planning. Injury.

[17] Oka K, Tanaka H, Okada K, Sahara W, Myoui A, Yamada T, Yamamoto M, Kurimoto S, Hirata H, Murase T (2019). Three-Dimensional Corrective Osteotomy for Malunited Fractures of the Upper Extremity Using Patient-Matched Instruments. J Bone Joint Surg Am.

[18] Chaouche S, Jacquet C, Fabre-Aubrespy M, Sharma A, Argenson J, Sebastien Parratte MO (2019). Patient-specific cutting guides for open-wedge high tibial osteotomy: safety and accuracy analysis of a hundred patients continuous cohort. Int Orthop.

[19] Roner S, Schweizer A, Da SY, Carrillo F, Nagy L, Fürnstahl P (2020). Accuracy and Early Clinical Outcome After 3-Dimensional Correction of Distal Radius Intra-Articular Malunions Using Patient-Specific Instruments. J Hand Surg Am.

[20] Wang C, Yu D, Xu C, Li M, Zhong D, Wang L, Liu H, Li Y (2021). Simulated operation combined with patient-specific instrumentation technology is superior to conventional technology for supramalleolar osteotomy: a retrospective comparative study. Am J Transl Res.

[21] Paley D, Herzenberg JE, Tetsworth K, Mckie J, Bhave A (1994). Deformity planning for frontal and sagittal plane corrective osteotomies. Orthop Clin North Am.

[22] Tanaka Y, Takakura Y, Hayashi K, Taniguchi A, Kumai T, Sugimoto K (2006). Low tibial osteotomy for varus-type osteoarthritis of the ankle. J Bone Joint Surg Br.

[23] Choi JY, Kim KW, Suh JS (2020). Low Tibial Valgization Osteotomy for More Severe Varus Ankle Arthritis. Foot Ankle Int.

[24] Wang H, Newman S, Wang J, Wang Q, Wang Q (2018). Corrective Osteotomies for Complex Intra-Articular Tibial Plateau Malunions using Three-Dimensional Virtual Planning and Novel Patient-Specific Guides. J Knee Surg.

[25] Mitchell EL, Furey P (2011). Prevention of radiation injury from medical imaging. J Vasc Surg.

[26] Biswas D, Bible JE, Bohan M, Simpson AK, Whang PG, Grauer JN (2009). Radiation exposure from musculoskeletal computerized tomographic scans. J Bone Joint Surg Am.

[27] Henckel J, Richards R, Lozhkin K, Harris S, Y FMR, Baena, Barrett ARW, Cobb JP. Very low-dose computed tomography for planning and outcome measurement in knee replacement the imperial knee protocol. J Bone Joint Surg Br. 2006;88(11):1513–8. 10.1302/0301-620X.88B11.17986 17075100

[28] Mao Y, Xiong Y, Li Q, Chen G, Fu W, Tang X, Yang L, Li J. 3D-Printed Patient-Specific Instrumentation Technique Vs. Conventional Technique in Medial Open Wedge High Tibial Osteotomy: A Prospective Comparative Study. Biomed Res Int. 2020;2020:1–10. 10.1155/2020/1923172 PMC7685795 33282939

