# Supplementary material for: Intra-articular opening wedge osteotomy for varus ankle arthritis with computer-assisted planning and patient-specific surgical guides: a retrospective case series
Source: BMC Musculoskelet Disord. 2022 May 21;23:483. doi: 10.1186/s12891-022-05437-z (PMC9123770; doi:10.1186/s12891-022-05437-z)
Supplement: Supplementary file 1 — Additional file 1: Table S1 The statistics for inter-observer reliability and intra-observer reproducibility [file 12891_2022_5437_MOESM1_ESM.doc]

Supplementary Table 1. The statistics for inter-observer reliability and intra-observer reproducibility

| Parameters | Inter-observer reliability | Intra-observer reproducibility |
| --- | --- | --- |
|  | ICC (95%CI) | ICC (95%CI) |
| TAS (^o^) | 0.843 (0.754-0.897) | 0.823 (0.705-0.914) |
| TT (^o^) | 0.989 (0.984-0.992) | 0.995 (0.993-0.996) |
| TMM (^o^) | 0.955 (0.924-0.972) | 0.950 (0.709-0.982) |
| TC (^o^) | 0.975 (0.964-0.983) | 0.977 (0.953-0.987) |
| TLS (^o^) | 0.886 (0.834-0.921) | 0.929 (0.898-0.950) |
| Distraction angle (^o^) | 0.979 (0.967-0.986) | 0.978 (0.965-0.988) |
| Distraction height (mm) | 0.945 (0.936-0.966) | 0.968 (0.945-0.989) |

**Abbreviations:** TAS: The tibial anterior surface angle; TT: The talar tilt angle; TMM: The tibial medial malleolus angle; TC: The talocrural angle; TLS: The tibial lateral surface angle; ICC: Intraclass correlation coefficients; CI, confidence interval.
